# Prevalence of Youth Overweight, Obesity, and Severe Obesity

**DOI:** 10.1001/jamanetworkopen.2025.58710

**Published:** 2026-02-10

**Authors:** William J. Heerman, Lauren R. Samuels, Jason P. Block, Keith A. Marsolo, Russell L. Rothman

**Affiliations:** 1 Department of Pediatrics at Vanderbilt University Medical Center, Nashville, Tennessee; 2 Department of Biostatistics at Vanderbilt University Medical Center, Nashville, Tennessee; 3 Department of Population Medicine, Harvard Pilgrim Health Care Institute, Harvard Medical School, Boston, Massachusetts; 4 Department of Population Health Sciences and the Duke Clinical Research Institute, Duke University School of Medicine, Durham, North Carolina; 5 Institute for Medicine and Public Health at Vanderbilt University Medical Center, Nashville, Tennessee

## Abstract

This cross-sectional study investigates the prevalence of overweight, obesity, and severe obesity among US youths aged 0 to 19 years in 2024.

## Introduction

Excess weight among youths is associated with an increased risk of adult obesity and short- and long-term health outcomes.^1,2^ Multiple US studies and surveillance programs have consistently documented high rates of youth obesity. However, national estimates for youth overweight and severe obesity remain limited, especially among subgroups. This study used electronic health records from a large national research network to provide updated estimates of youth overweight, obesity, and severe obesity.

## Methods

This cross-sectional study used data from the National Patient-Centered Clinical Research Network (PCORnet), which includes 8 clinical research networks and was used previously for US population health surveillance.^3,4^ In June 2025, PCORnet issued an electronic query to characterize patients in its network for 2024. Queries were executed locally against each site’s PCORnet common data model, and aggregate, deidentified results were returned. PCORnet does not maintain a centralized patient-level data repository.

The query included youths (ages 0-19 years) with a recorded height and weight within 14 days of each other in 2024. Age- and sex-specific body mass index (BMI; calculated as weight in kilograms divided by height in meters squared) percentiles were calculated. For children younger than 2 years, categories were based on World Health Organization standards (<2.3rd, 2.3rd to <97.7th, 97.7th to <99th, and ≥99th percentile). For youths aged 2 years to younger than 19 years, categories were based on Centers for Disease Control and Prevention standards for underweight, healthy weight, overweight, obesity, and severe obesity (<5th, 5th to <85th, 85th to <95th, 95th to <120% of the 95th, and ≥120% of the 95th percentile, respectively). PCORnet uses a method to remove implausible height and weight values.^5^ No missing values were imputed. The Vanderbilt University Medical Center Institutional Review Board classified this as non–human participants research and so exempt from review and informed consent. We followed the STROBE reporting guideline.

## Results

Data were available for 6 094 825 youths (49.1% female; 2 437 173 aged 12-19 years [40.0%]; 0.7% American Indian or Alaska Native, 5.2% Asian, 17.0% Black, 0.3% Native Hawaiian or Other Pacific Islander; 55.1% White, 2.6% multiple races, 7.9% other race, and 11.2% missing race; 24.1% Hispanic or Latino) across the US, with broad geographic representation. Among youths ages 2 to 19 years, 19.8% had obesity. In early childhood (ages 2-5 years), 26.9% had overweight or obesity, increasing to 38.5% in adolescence and young adulthood (ages 12-19 years). Severe obesity occurred among 9.2% of adolescents and young adults. The Figure and Table show prevalence by age, sex, race, and ethnicity. Among ages 12 to 19 years, healthy weight was found in 49.5% of American Indian or Alaska Native, 52.3% of Black, 49.1% of Hispanic, 47.3% of Native Hawaiian or Other Pacific Islander, and 59.3% of White youths.

**Figure.**
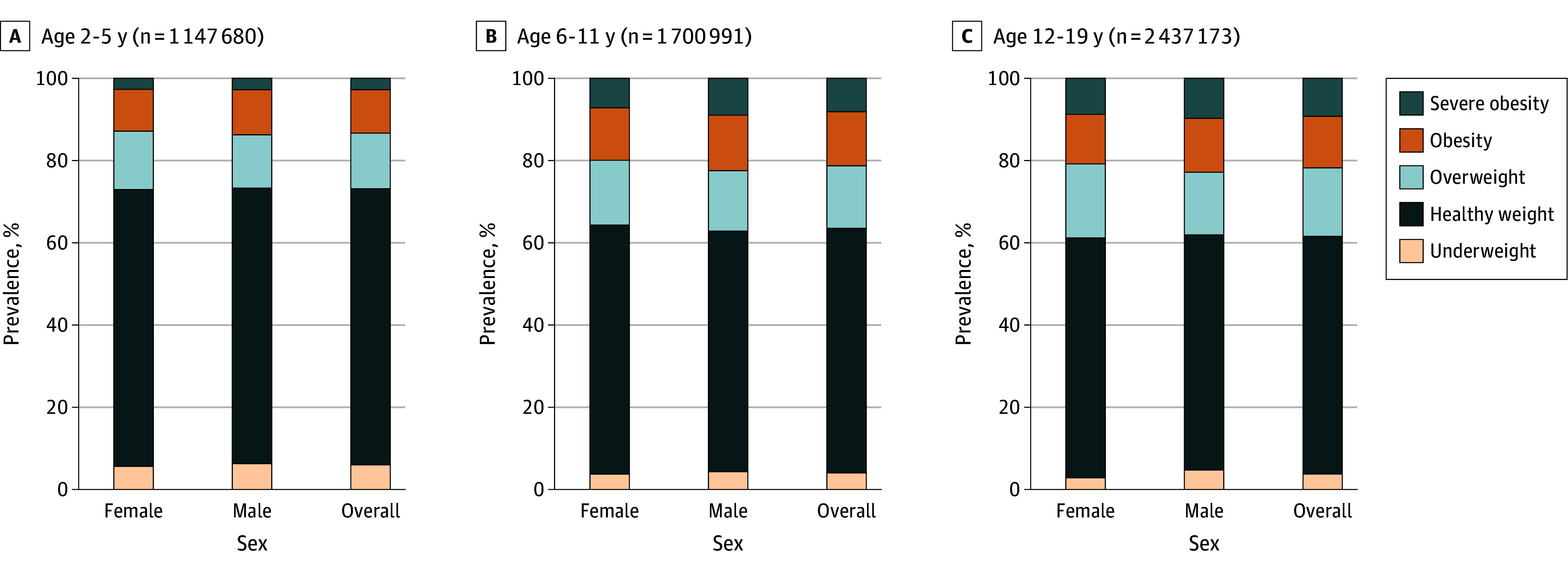
Prevalence of Youth Weight Status by Age and Sex

**Table.** Prevalence of Youth Weight Status by Age and Race and Age and Ethnicity^a^

Weight status^b^	Youths, No. (%) (N = 6 094 825)											
	American Indian or Alaska Native	Asian	Black or African American	Native Hawaiian or Other Pacific Islander	White	Multiple races	Other race	Missing race	Not Hispanic	Hispanic	Other ethnicity	Missing ethnicity
**Ages 0-1 y**												
Youths, No.	5604	44 140	142 625	2839	394 695	27 191	64 775	127 112	462 168	198 395	9447	138 971
<2.3rd Percentile	258 (4.6)	4413 (10.0)	15 275 (10.7)	121 (4.3)	33 760 (8.6)	885 (3.3)	3747 (5.8)	11 945 (9.4)	27 206 (5.9)	8699 (4.4)	278 (2.9)	34 221 (24.6)
2.3rd to <97.7th Percentile	4648 (82.9)	37 259 (84.4)	112 940 (79.2)	2397 (84.4)	327 496 (83.0)	24 193 (89.0)	53 166 (82.1)	101 572 (79.9)	395 790 (85.6)	166 420 (83.9)	8535 (90.3)	92 926 (66.9)
97.7th to <99th Percentile	186 (3.3)	795 (1.8)	4340 (3.0)	92 (3.2)	11 987 (3.0)	782 (2.9)	2206 (3.4)	3882 (3.1)	13 322 (2.9)	7473 (3.8)	248 (2.6)	3227 (2.3)
≥99th Percentile	512 (9.1)	1673 (3.8)	10 070 (7.1)	229 (8.1)	21 452 (5.4)	1331 (4.9)	5656 (8.7)	9713 (7.6)	25 850 (5.6)	15 803 (8.0)	386 (4.1)	8597 (6.2)
**Ages 2-5 y**												
Youths, No.	8200	63 248	207 582	3762	605 954	35 627	96 689	126 618	730 082	290 759	11 289	115 550
Underweight	441 (5.4)	6993 (11.1)	13 715 (6.6)	181 (4.8)	32 218 (5.3)	2039 (5.7)	5456 (5.6)	7817 (6.2)	47 261 (6.5)	13 326 (4.6)	697 (6.2)	7576 (6.6)
Healthy weight	4984 (60.8)	45 469 (71.9)	135 564 (65.3)	2296 (61.0)	414 328 (68.4)	24 224 (68.0)	61 515 (63.6)	82 168 (64.9)	507 201 (69.5)	177 617 (61.1)	8136 (72.1)	77 594 (67.2)
Overweight	1240 (15.1)	5880 (9.3)	28 587 (13.8)	598 (15.9)	83 463 (13.8)	4702 (13.2)	13 524 (14.0)	17 527 (13.8)	94 456 (12.9)	44 156 (15.2)	1384 (12.3)	15 525 (13.4)
Obesity	1176 (14.3)	4052 (6.4)	23 473 (11.3)	513 (13.6)	61 159 (10.1)	3739 (10.5)	12 346 (12.8)	14 859 (11.7)	66 442 (9.1)	42 216 (14.5)	889 (7.9)	11 770 (10.2)
Severe obesity	359 (4.4)	854 (1.4)	6243 (3.0)	174 (4.6)	14 786 (2.4)	923 (2.6)	3848 (4.0)	4247 (3.4)	14 722 (2.0)	13 444 (4.6)	183 (1.6)	3085 (2.7)
**Ages 6-11 y**												
Youths, No.	11 581	94 750	286 383	5401	943 314	44 020	140 788	174 754	1 109 080	416 706	15 401	159 804
Underweight	364 (3.1)	7379 (7.8)	9738 (3.4)	169 (3.1)	37 254 (3.9)	1801 (4.1)	5253 (3.7)	7070 (4.0)	49 034 (4.4)	12 123 (2.9)	776 (5.0)	7095 (4.4)
Healthy weight	5952 (51.4)	62 708 (66.2)	156 569 (54.7)	2682 (49.7)	581 841 (61.7)	26 031 (59.1)	76 764 (54.5)	99 000 (56.7)	697 549 (62.9)	208 175 (50.0)	10 073 (65.4)	95 750 (59.9)
Overweight	1984 (17.1)	12 641 (13.3)	46 159 (16.1)	940 (17.4)	140 430 (14.9)	6608 (15.0)	22 412 (15.9)	27 442 (15.7)	161 375 (14.6)	71 220 (17.1)	2193 (14.2)	23 828 (14.9)
Obesity	1941 (16.8)	8848 (9.3)	41 817 (14.6)	915 (16.9)	117 366 (12.4)	5794 (13.2)	21 889 (15.5)	25 396 (14.5)	127 432 (11.5)	74 680 (17.9)	1574 (10.2)	20 280 (12.7)
Severe obesity	1340 (11.6)	3174 (3.3)	32 100 (11.2)	695 (12.9)	66 423 (7.0)	3786 (8.6)	14 470 (10.3)	15 846 (9.1)	73 690 (6.6)	50 508 (12.1)	785 (5.1)	12 851 (8.0)
**Ages 12-19 y**												
Youths, No.	17 168	116 112	400 606	7282	1 413 057	50 739	178 757	253 452	1 624 165	564 583	19 649	228 776
Underweight	493 (2.9)	6907 (5.9)	13 110 (3.3)	191 (2.6)	53 587 (3.8)	1849 (3.6)	6278 (3.5)	10 086 (4.0)	64 551 (4.0)	16 192 (2.9)	854 (4.3)	10 904 (4.8)
Healthy weight	8503 (49.5)	78 686 (67.8)	209 620 (52.3)	3446 (47.3)	838 123 (59.3)	28 510 (56.2)	95 549 (53.5)	145 262 (57.3)	980 244 (60.4)	277 395 (49.1)	12 958 (65.9)	137 102 (59.9)
Overweight	3256 (19)	16 773 (14.4)	68 365 (17.1)	1382 (19)	232 292 (16.4)	8571 (16.9)	31 596 (17.7)	43 833 (17.3)	257 692 (15.9)	108 680 (19.2)	2895 (14.7)	36 801 (16.1)
Obesity	2741 (16)	9654 (8.3)	55 183 (13.8)	1166 (16)	171 727 (12.2)	6601 (13.0)	26 079 (14.6)	32 744 (12.9)	184 245 (11.3)	93 924 (16.6)	1852 (9.4)	25 874 (11.3)
Severe obesity	2175 (12.7)	4092 (3.5)	54 328 (13.6)	1097 (15.1)	117 328 (8.3)	5208 (10.3)	19 255 (10.8)	21 527 (8.5)	137 433 (8.5)	68 392 (12.1)	1090 (5.5)	18 095 (7.9)

^a^ Participant race and ethnicity were based on data from the electronic health record, which may have variable sources across clinical practices included in this analysis. Race and ethnicity were included because obesity prevalence differs across these groups and reporting these differences provides a more complete description of the study population. In National Patient-Centered Clinical Research Network (PCORnet) data, race and Hispanic ethnicity are stored as 2 separate variables. The first 8 columns show options for the race variable from the dataset, with *missing* combining the options *refuse to answer*, *no information*, and *unknown*. The last 4 columns reflect options for the Hispanic ethnicity from the dataset of *yes*, *no*, and *other*, with *missing* combining the options *refuse to answer*, *no information*, and *unknown*.

^b^ Weight statuses are based on age- and sex-specific body mass index percentiles calculated using World Health Organization standards for ages 0 to 1 year and Centers for Disease Control and Prevention standards for ages 2 to 19 years.

## Discussion

This cross-sectional study found that in 2024, excess youth overweight and obesity remained highly prevalent among youths in the US. The prevalence of excess adiposity was higher for certain racial and ethnic subgroups. Among children younger than 2 years, prevalences of BMI in the 99th percentile or greater were between 3.8% and 9.1%. The overall prevalence of youth obesity characterized through PCORnet data resources is consistent with 2025 prevalence data from the National Health and Nutrition Examination Survey (NHANES),^6^ suggesting that PCORnet data resources may be appropriate for future studies of youth overweight and obesity. A specific advantage may be the potential for data that are representative of the US population. This study provides additional details about the prevalence of youth overweight and subgroups that have been previously underrepresented (eg, Asian and American Indian or Alaska Native youths).

Limitations include that data came from electronic health records, so many youths did not have a BMI measure. Additionally, these estimates were not weighted. These results demonstrate the uneven distribution of obesity and severe obesity across youths in the US and underscore the need for ongoing treatment, prevention, and public health interventions to reduce excess adiposity in youths.

## Notes

Supplement.
Data Sharing Statement
