# Supplementary material for: Prevalence of Youth Overweight, Obesity, and Severe Obesity
Source: JAMA Netw Open. 2026 Feb 10;9(2):e2558710. doi: 10.1001/jamanetworkopen.2025.58710 (PMC12892151; doi:10.1001/jamanetworkopen.2025.58710)
Supplement: Supplement. — Data Sharing Statement [file jamanetwopen-e2558710-s001.pdf]

Data Sharing Statement

Heerman. Prevalence of Youth Overweight, Obesity, and Severe Obesity. *JAMA Netw Open*. Published online February 10, 2026. doi:10.1001/jamanetworkopen.2025.58710

Data
Data available: No

Additional Information
Explanation for why data not available: Publicly avialable data
